# Integrating psychiatry into a community based palliative care team for structurally vulnerable populations: a descriptive retrospective cohort study of the PEACH psychiatry program model

**DOI:** 10.1186/s12904-026-01996-3

**Published:** 2026-01-21

**Authors:** Ovini Thomas, Jude Sanon, Lauren Thomson, Naheed Dosani, Daniel Rosenbaum, Trevor Morey

**Affiliations:** 1 Temerty Faculty of Medicine, University of Toronto, Toronto, ON Canada; 2 Palliative Education And Care for the Homeless (PEACH), Inner City Health Associates, Toronto, ON Canada; 3 MAP Centre for Urban Health Solutions, St. Michael’s Hospital, Unity Health Toronto, Toronto, ON Canada; 4 Department of Family and Community Medicine, St Michael’s Hospital, Unity Health Toronto, Toronto, ON Canada; 5 Department of Family and Community Medicine, University of Toronto, Toronto, ON Canada; 6 University Health Network, Toronto, ON Canada; 7 Temmy Latner Centre for Palliative Care, Mount Sinai, Hospital, 60 murray st, 4th floor, Toronto, ON M5T 3L9 Canada

**Keywords:** Palliative care, Mental illness, Psychiatry care, Homelessness, Structural vulnerabilities, Integrated care, End of life care, Health equity, Psychiatry

## Abstract

**Background:**

Structurally vulnerable populations have higher morbidity rates and lower life expectancy than the general population. Additionally, there is a high prevalence of mental illness and substance use disorders within this group, which compounds their health issues and adds complexity to providing palliative care. The Palliative Education and Care for the Homeless (PEACH) program in Toronto provides palliative care to this population, and includes an integrated psychiatry service to support patients with mental illness. The aim of this paper is to describe a novel model of integrated psychiatric care and palliative care for structurally vulnerable populations living with life-threatening illness and complex mental health needs.

**Methods:**

We conducted a retrospective cohort study, collecting data from all patients referred to the PEACH psychiatry service in Toronto, Ontario, Canada from 2019 to 2023. The study included patients currently followed by PEACH Psychiatry, those deceased or discharged, and those referred but not assessed. Descriptive statistics were used to analyze the data.

**Results:**

Out of 62 patients referred to PEACH psychiatry, 48 were included in the analysis. The majority were male (77.1%) and were aged 50–70 (68.7%). Referrals were primarily for psychiatric symptom management (45.8%) and support around coping with life-threatening illness (22.9%). Half of the patients had two or more psychiatric diagnoses, with depressive disorders, substance use disorder, and psychotic disorders being most common. Substance use disorder was the most common diagnostic impression post-assessment by psychiatry (25.0%). Therapies provided included medication management, psychotherapy, and diagnostic clarification.

**Conclusions:**

This study outlines a model of integrated psychiatric care within a community-based palliative team serving structurally vulnerable populations. This kind of integrated care represents an important service to improve delivery of both palliative care and psychiatric support for structurally vulnerable populations.

**Supplementary Information:**

The online version contains supplementary material available at 10.1186/s12904-026-01996-3.

## Background

Structurally vulnerable populations experience higher morbidity rates and have a markedly lower life expectancy compared to the general population. Structural vulnerability refers to a population group’s condition of being at risk for negative health outcomes through their interface with socioeconomic, political and cultural/normative hierarchies [1]. This includes those experiencing homelessness [2], living in poverty, gaps in service access, a mistrust of the healthcare system, provider bias and challenges receiving appropriate care [1–6]. Additionally, the prevalence of mental health and substance use disorders (SUDs) are disproportionately high among this population, compounding their health issues [4, 7, 8]. Individuals with a life-threatening illness and severe and persistent mental illness (SPMI) [9] face additional barriers, including stigma, social isolation, and difficulties with information processing and communication. These challenges hinder their access to appropriate advance care planning and palliative care [10–14]. People with SPMI have higher mortality, are less likely to receive treatment after a cancer diagnosis, and are less likely to receive opioid analgesia in the last months of life [15, 16].

Previous literature has described the challenges of providing palliative care for people with mental illness as well as SUDs [15, 17], with a common issue being the lack of coordinated care between mental health clinicians, primary care and palliative care providers. Services are often ‘siloed’, which can prevent good collaborative care between psychiatric and palliative care providers. Furthermore, literature exploring how best to provide physical and mental health care to people living with mental illness suggests that integrating these services reduces barriers to care; however, this is unfortunately the exception rather than the norm for providing care to people with mental illness [17–23].

High-quality psychosocial care is a key element of holistic palliative care [24]. However, there are well-documented barriers for people with structural vulnerabilities in accessing mainstream psychosocial oncology services. Previous literature has outlined strategies to address these gaps and barriers, including by enhancing flexibility of care delivery (e.g., by offering community-based care), targeting treatment approaches for specific populations, and expanding care teams to include community health workers and health navigators [25].

Studies have also highlighted that even for mainstream palliative care services, the mental health issues of some patients can exceed the available resources of palliative care teams requiring the expertise of psychologists or psychiatrists. In models where psychologists or psychiatrists are core members of the care team, patient well-being is noted to improve [26].

Despite broad recognition of these challenges, there is a lack of literature that adequately describes or evaluates models of care for people living with life-threatening illness, mental illness, and SUDs. This “tri-morbidity” - along with the added barrier of structural vulnerability - presents unique obstacles in delivering high-quality palliative care, and the social and health inequities these individuals face often exacerbate their psychological distress in the face of life-threatening illness [7]. In recognition of the barriers to accessing high quality palliative care for structurally vulnerable populations a number of specialized community based programs have been developed to help improve equitable access to care [27–29].

Palliative Education and Care for the Homeless (PEACH) is a community-based outreach palliative care program, established in 2014, that provides palliative care to individuals facing structural vulnerabilities through a harm reduction and trauma informed approach in Toronto, Ontario, Canada. PEACH is housed within Toronto’s Inner City Health Associates, a homeless health services organization, and is staffed by palliative care physicians, a psychiatrist, a virtual nurse coordinator, a health navigator, a dedicated outreach nurse, and a peer worker. The team collaborates closely with publicly funded home care providers (e.g., PSWs, nurses, and other allied health workers).

A retrospective chart review of PEACH indicated that 41.3% of patients referred to the program between 2014 and 2017 had at least one documented mental health diagnosis, and 41.3% reported using one substance [4]. These patients have complex psychosocial needs that are not always addressed by mainstream care models, and they face significant barriers to accessing care due to stigma, discrimination, and mobility challenges. Recognizing the barriers faced by patients with mental illness, the psychiatry arm of PEACH was introduced in 2019.

Prior to the formation of the integrated PEACH psychiatry model, community psychiatric consultation was disjointed and difficult to access in a timely manner. Given the limited prognosis and low functional status of some PEACH patients, they would often become too unwell or die prior to receiving a comprehensive psychiatric assessment, let alone psychotherapeutic intervention. Furthermore, timely psychiatric assessment would often be limited to hospital-based psychiatrists, and given the previous negative experiences of many patients in these types of healthcare environments this would often be seen as unacceptable for them. From time to time, there was access to on-the-fly “hallway consult” support, but these encounters were few and far between. It was felt that an integrated outreach model that could provide longitudinal care in a community setting would help to provide timely access to psychiatric support for PEACH patients while meeting them where they are at in the community.

In this paper we describe the novel, integrated model of care that addresses both palliative and psychiatric care needs for structurally vulnerable patients in Toronto, Ontario, Canada living with a life-threatening illness. Key objectives of this study include describing the cohort of patients served, reasons for referral, and interventions provided by the service. We hope that this description of the PEACH psychiatry service will offer insight for other palliative care programs and improve access to high-quality palliative care and psychiatric care for structurally vulnerable populations.

## Methods

### Study population

This retrospective cohort study included all patients who were referred to and received care from the PEACH psychiatry team in Toronto, Ontario, Canada from January 2019 to May 2023. The study population included those who are currently followed by the PEACH psychiatry team, those who are now deceased or have been discharged, and those who were referred but never assessed.

### Setting: the PEACH psychiatry service

The PEACH integrated psychiatry service was introduced in 2019. This initiative sought to address the complex psychosocial needs of patients with tri-morbidity by offering integrated, mobile psychiatric care for interested patients. PEACH also provides collaborative care with mainstream psychiatry services like hospital-based psychosocial oncology departments and Assertive Community Treatment (ACT) teams; however, an integrated model of care was piloted to improve access to psychiatric care for those who have not been connected with a psychiatrist or other mental health supports. The psychiatry service is funded through the Inner City Health Associates, the same organization that provides funding for PEACH, and it offers one day per week of outreach by a psychiatrist along with senior psychiatry resident physicians.

Alongside formal psychiatric consultation and follow-up care, the PEACH psychiatrist provides informal consultation to team members in cases around appropriateness of potential patient referrals and in cases where patients decline psychiatry involvement. The service also provides mentorship and coaching to PEACH team members around psychosocial aspects of palliative care for patients with mental illness. Education, coaching, and support are offered in monthly team rounds, as well as informally on an as-needed basis. The PEACH psychiatrist also helps to collaborate with inpatient psychiatrists during hospital admissions for PEACH patients to ensure continuity of care. The scheduling of PEACH psychiatry visits is supported by the PEACH team’s virtual nurse coordinator. Joint visits between the psychiatrist and the palliative care physician sometimes occur, though it is more common for joint visits to occur between the psychiatrist and other interdisciplinary PEACH team members such as the PEACH health navigator, PEACH nurse, the collaborating home care coordinator, or other community support workers (e.g., housing workers, addictions workers, etc.).

Additionally, the psychiatry team is available by phone and by email beyond scheduled outreach, ensuring continuity of care. Because PEACH is a mobile palliative care service (i.e., there is no dedicated clinical space), the PEACH psychiatry arm cannot be described as “co-located”. However, the psychiatry service is integrated in several other ways, including a shared Electronic Medical Record; attendance at monthly team operations meetings; inclusion in daily email communications from palliative care clinicians about their clinical encounters; and facilitation of monthly rounds with allied health members of the PEACH team which focus on patients followed by the psychiatry service. PEACH psychiatry also provides liaison services (i.e., connection with hospital or hospice staff, patient visits at these care settings, and participation in interdisciplinary case conferences). In these ways, PEACH psychiatry meets many of the nine organizational and structural arrangements for good integrated care for people with mental health problems identified by Rodgers et al. [18], which are as follows: 1. Information sharing systems, 2. Shared protocols, 3. Joint funding and commissioning, 4. Co-location of services (e.g., services brought together for physical and practical ease of access), 5. Multidisciplinary teams, 6. Liaison services (e.g., provision of shared expertise, across service settings), 7. Navigators (e.g., named care co-ordinators), 8. Reduction of stigma, 9. Research (e.g., to ascertain the best way of delivering and evaluating integrated care).

Because PEACH is a mobile palliative care service, a provider experienced with outreach-based community psychiatry was sought from the outset. The following skills and experience were also sought: comfort working with patients with SPMI and those who use drugs; experience with medical psychiatry, including specifically the psychotherapeutic care of patients facing advanced disease; and comfort working in a multidisciplinary team. The first psychiatrist who occupied the PEACH psychiatry role (DR) had completed specialized training in both Assertive Community Treatment psychiatry in the inner city setting, as well as in psychosocial oncology and palliative care, including formal certification in Managing Cancer and Living Meaningfully (CALM) Therapy, a brief, individual psychotherapy developed to relieve distress and improve quality of life among individuals facing advanced cancer [30]. In some ways, the PEACH psychiatry service reflects a blending of these worlds (i.e., outreach-based psychiatric care for patients with SPMI and structural vulnerabilities together with psychotherapeutic care of patients with advanced disease).

### Cohort description at time of initial palliative care consultation

Descriptive statistics such as age, gender, palliative performance status (PPS), palliative care diagnosis, housing status, previous psychiatric diagnoses and substance use history were collected retrospectively from the initial palliative care consultation. At initial palliative care consultation patients discuss their code status with the physician (i.e. “full resuscitation” vs. “Do not resuscitate”); this was also collected at the time of initial palliative care consultation from the electronic medical record.

### Description of psychiatric services provided

Information on the reason for referral to psychiatry services and time to initial psychiatric consultation were collected retrospectively at the time of initial psychiatry consultation. Measures including the length of the therapeutic relationship with the psychiatry team, diagnostic impression, visit type (virtual vs. outreach), therapies offered, and reason for discontinuing services were collected retrospectively from the time of psychiatric consultation.

### Data collection and analysis

All data were collected retrospectively from the electronic medical record. Descriptive statistics were used to describe the cohort, and for confidentiality we have not reported any small cell sizes (*N* < 3). Data are reported in frequency along with percentage of the cohort. Data were verified by 3 separate members of the team for accuracy. Research ethics approval was obtained from the St.Michael’s Hospital Research Ethics Board (REB#23 − 004).

## Results

### Demographics

From January 2019 to May 2023, a total of 62 patients were referred to the PEACH Psychiatry team. Thirteen patients were excluded from the study as they were not formally seen by the psychiatry team, or had declined palliative care services. Among the 48 patients included in this dataset, the majority identified as male (77.1%), were aged 50 to 70 (68.7%) and resided in subsidized housing (43.7%) or shelter (31.3%) at time of referral to psychiatry. See Table 1 for details.

**Table 1:** Core philosophies of PEACH psychiatry service

Core Philosophies of PEACH Psychiatry Service	
Trauma informed lens	Aims to reduce stigma and barriers to care faced by many PEACH patients by virtue of their structural vulnerabilities.
Outreach	Community-based outreach model of care that meets people where they are at
Low barrier for referral	Patients do not need to have a psychiatric diagnosis to be referred to the service, nor is there a requirement for suspicion of a psychiatric diagnosis. Holding close the maxim that “no one knows how to have cancer (or other life-limiting illness), and everyone who desires extra support deserves it”
Normalizing approach	Our approach seeks to normalize and validate experiences of distress in the face of advanced disease, death/dying, and continued experiences of marginalization and poverty.
Prioritizing trust	Many patients who may benefit from and deserve robust psychological support in the face of life-threatening illness have also had experiences of coercive care and trauma in institutional settings. A gentle, non-judgmental approach is taken, striving to meet patients where they’re at in terms of possible ambivalence about seeing a psychiatrist. Patient engagement, trust, and relationship-building are prioritized.
Longitudinal care	The capacity and flexibility to provide timely access to longitudinal care is crucial.
Harm reduction	Working collaboratively with patients to solicit values and help them define goals of care both with respect to their medical illness and related treatment, and also with respect to their substance use (i.e., supporting harm reduction)

### Referral information

Patients were primarily referred for assistance with psychiatric symptom management (45.8%), while many others were referred for support around coping with a life-threatening illness (22.9%). Many patients were referred to psychiatry within 1 week of palliative care consultation (31.3%); however, a majority of patients were referred 12 weeks or longer after initial involvement with the palliative care team (39.6%). patients were typically seen by the psychiatry service within 1–4 weeks (58.3%) of referral from the palliative care physician. 50% of the patients had two or more previous psychiatric diagnoses; depressive, substance use, and psychotic disorders were the most common prior diagnoses. Most patients had a substance use disorder of 1 or more substances (56.3%). Patients referred to psychiatry typically had a Palliative Performance Scale score of > 60% (66.7%), and the majority had malignancy as a primary palliative diagnosis (60.4%). See Table 2 for details.

**Table 2:** Demographics of patients Seen by PEACH Psychiatry Team

Variable	Characteristics	Frequency (*n*=48)	Percent
Age	20 - 39	3	6.3%
	40 - 49	6	12.5%
	50 - 59	16	33.3%
	60 - 69	17	35.4%
	70+	6	12.5%
Sex	M	37	77.1%
	F	11	22.9%
	NB	0	0.0%
Palliative Diagnosis	Malignant	29	60.4%
	Non-malignant	14	29.2%
	Infection	5	10.4%
Housing Status	Subsidized-Community Housing	21	43.7%
	Shelter	15	31.3%
	Market Rent	3	6.3%
	Transitional / Supportive Housing	6	12.5%
	Other (no fixed address, hospital, sleeping rough, couchsurfing).	3	6.3%
Code Status at initial PEACH Palliative Care Encounter	Full Code	36	75.0%
	DNR/DNI	12	25.0%
Palliative Performance Scale	<30%	0	0.0%
	40-50%	16	33.3%
	60%	15	31.3%
	70%+	17	35.4%
Substance Use Disorders	0 (no substance use)	16	33.3%
	1 (a single substance use disorder)	15	31.3%
	2+ (polysubstance use disorder)	12	25.0%
	Recreational use, not meeting criteria for substance use disorder	5	10.4%
Previous Psychiatric Diagnoses	None	10	20.8%
	1	14	29.2%
	2+	24	50.0%

### Psychiatry therapeutic relationship

The majority of patients referred to psychiatry were seen 2–4 times (41.7%); however, 11 patients were seen over 10 times (22.9%). Most patients (68.7%) were seen exclusively by in-person community visits, and 35.4% of patients were followed for over twelve months.

### Therapies/services provided by the psychiatric team

The most common diagnostic impression after psychiatric involvement was substance use disorder (25.0%) followed by an equal split between delirium/neurocognitive disorders (16.6%), mood disorders (16.6%), and 16.6% of patients with either normative distress or with an unclear diagnostic picture. The therapies provided by the psychiatry team included psychotropic medication modification/initiation and psychotherapy (41.7%), psychotherapy alone (22.9%), supportive counselling (14.6%) and diagnostic clarification (12.5%). Four patients (8.3%) had limited assessment and did not have any therapies provided. Death was the most common reason for discontinuing psychiatry services (37.5%). Nine patients (18.8%) were seen for a one time consultation. Otherwise, the psychiatric relationship remains ongoing for 18 patients (36.7%). See Table 3 for details.

**Table 3:** Psychiatric Services Provided by PEACH Psychiatry Team and Reason for Referral

Variable	Characteristic	Frequency (*n*=48)	Percent
Reason for referral	Psychiatric symptoms (Depression, psychosis, ect)	22	45.8%
	Counseling to support coping with a serious illness	11	22.9%
	Memory or other cognitive dysfunction	4	8.3%
	Capacity issues	3	6.3%
	Psychiatric medication assessment	3	6.3%
	Other: Support for care team due to patient complexity, decision making difficulties, Problematic substance use, Concern about patient ability to care for self, Suicide or self harm risk	5	10.4%
Time from initial palliative care consult to psychiatry referral	<1 week	15	31.3%
	1 - 4 weeks	9	18.8%
	4 - 12 weeks	5	10.4%
	12 - 26 weeks	11	22.9%
	>26 weeks	8	16.7%
Time from referral to psychiatry to first psychiatric visit	<1 week	13	27.1%
	1-4 weeks	28	58.3%
	4-12 weeks	7	14.6%
Visits per patient	1 visit	10	20.8%
	2 - 4 visits	20	41.7%
	5 - 9 visits	7	14.6%
	10+ visits	11	22.9%
Type of visit	Primarily virtual	4	8.3%
	Primarily outreach/ half/half	11	22.9%
	Exclusively outreach	33	68.8%
Length of relationship	<1 month	9	18.8%
	1 - 3 months	5	10.4%
	3 - 6 months	10	20.8%
	6 - 12 months	7	14.6%
	>12 months	17	35.4%
Diagnostic Impression after Psychiatric Involvement	Substance Use Disorder	12	25.0%
	Delirium/ Neurocognitive Disorder	8	16.6%
	Mood disorders (depression, bipolar)	8	16.6%
	None/diagnosis not yet clear/ normative grief	8	16.6%
	Psychotic disorders (psychosis, schizophrenia, schizoaffective)	5	10.4%
	Trauma-related disorders (PTSD)	4	8.3%
	Anxiety disorders	3	6.3%
Therapies Provided	Psychotropic Medication modification/initiation + Psychotherapy	20	41.7%
	Psychotherapy alone	11	22.9%
	Supportive counseling	7	14.6%
	Diagnostic clarification	6	12.5%
	Limited assessment	4	8.3%
Reason for discontinuing psychiatry services	Patient died	18	37.5%
	Relationship ongoing	18	37.5%
	One time consultation	9	18.8%
	Other: Transferred to hospital/hospice, Patient Declined further services, Unknown	3	6.3%

## Discussion

### Key findings

This study describes a novel model of integrated psychiatric care for a community-based palliative care team that serves structurally vulnerable populations. Most patients are seen in an exclusively outreach model and are offered both psychotherapy and psychotropic medication management. Only one patient declined further services after initial psychiatric consultation; for most patients, services were only discontinued after they were transferred to hospice or died. Patients were referred quickly, most within 1 week, after assessment by a palliative care physician. However, it is also notable that many patients were referred to psychiatry only after being followed by the palliative care physician for a number of months. Many patients may not be agreeable to a referral to a psychiatrist until enough rapport is built with the team despite having significant psychiatric symptoms. In some cases patients may not disclose psychiatric symptoms until they have built trust with their care team, which may be several months after initial palliative care consultation. This finding may also be attributed to the transient nature of a structurally vulnerable population who are also living with a life threatening illness who frequently change living environments due to unstable housing and have prolonged hospitalizations leading to periodic gaps in care. Most patients received services for 2–4 visits and some patients had longitudinal follow-up over 12 months, emphasizing the importance of early referrals to maximize the impact of psychiatric interventions in palliative care.

Our results suggest that the role of psychiatry in palliative care for structurally vulnerable populations has a broad scope, ranging from diagnostic clarification, treatment recommendations, ongoing support and psychotherapy, informal support for the care team, liaison with inpatient providers, and one-off consultation to aid with capacity assessment. This integrated model allows for patients living with mental illness and those without mental illness who need psychosocial support to have access to both psychiatric care and symptom management when living with a life-threatening illness.

Given the high prevalence of patients with substance use disorders (SUDs) in our study sample, it is important to note that addictions care is provided primarily by the PEACH palliative care MDs - some of whom have specialized training in addictions medicine - and sometimes in collaboration with other addictions medicine specialists separate from the PEACH psychiatry team. Although many patients received a primary psychiatric diagnosis of substance use disorder alone after initial assessment with the PEACH psychiatry team (25%), the reason for referral to the psychiatry service was for psychiatric symptoms or coping with a life-threatening illness and not for management of substance use issues. We do note that in many other models of care psychiatrists would likely be helping to manage substance use disorders more directly and may represent another potential use case for an integrated psychiatry model. It is also notable that a relatively high percentage (16.6%) did not receive a psychiatric diagnosis after initial assessment but rather were felt to either not have a clear diagnosis or be experiencing normative emotional reactions to life-limiting illness together with the stressors associated with poverty, food insecurity, unstable housing, and related structural factors.

### What this study adds

Previous literature has highlighted that collaborations between psychiatry and palliative care can be challenging to achieve through mainstream services [16–23]. Additionally, recent systematic reviews of palliative care models for homeless adults [31, 32] have highlighted the importance of integrated care to ensure access to high quality palliative care for structurally vulnerable populations, with one review highlighting that the most common recommendation for ensuring access to palliative care was to integrate palliative care and social service provision. Importantly, one review noted that embedded mental and physical health care, social programming, and spiritual care were seen as important for palliative care provision in this patient population [31]. While previous studies have shown that integrated psychiatry and physical health services can lead to improved vaccination rates and chronic disease screening for people living with mental illness [18], no previous studies have described a model of integrated psychiatry and palliative care for structurally vulnerable populations. There have been significant strides made to develop community based palliative care teams for structurally vulnerable populations across Canada with varying levels of integration with social service providers; [27–29] while these programs help to provide access to palliative care for this population, only the PEACH program has an integrated psychiatry model at this time.

The PEACH integrated psychiatry model takes a unique approach of integrating community based psychiatry with community based palliative care, reducing barriers for structurally vulnerable patients with life-threatening illness to receive psychiatric services. We hope this study will serve as a useful example for community-based palliative care teams caring for structurally vulnerable populations on how to provide integrated psychiatric care for their patients.

### Strengths and weaknesses/limitations

This study’s strength is that it is the first to our knowledge to describe a model of integrated psychiatric and community based palliative care for structurally vulnerable populations.

The limitations of the study include the lack of healthcare utilization data and patient reported outcome measures for this cohort. Future studies should evaluate whether this model has an impact on mental health specific patient reported outcome measures, such as the PHQ-9 [33], however, this would also require a team to routinely collect this data during psychiatry visits. Additionally, the study’s quantitative nature leaves gaps in understanding the qualitative impact on patients and providers. Finally, this study cannot make any conclusions about the impact of the PEACH psychiatry service, including its acceptability, given that the data are descriptive and do not include outcomes or patient perspectives.

Because the PEACH psychiatry service represents a model of integrated care, another limitation of this study is that we have not described patients followed by PEACH that receive psychiatric care from other community psychiatry teams. This is often the case for patients with SPMI who may have close relationships with, e.g., Assertive Community Treatment or similar teams. In an effort to enhance access to both mental health and palliative care for patients with SPMI, there may be a tension between prioritizing existing relationships – which in some cases are deep and longstanding – and the virtues of integrated mental health and palliative care (e.g., shared information systems, co-location, etc.). Indeed, depending on the needs of the patient, a more traditional community psychiatry team may be best suited to provide mental health care in collaboration with a separate community palliative care team. This might be the case, for example, if there is a need for psychiatric case management, supervised medication administration, or mandatory outpatient treatment.

### Next steps

A growing network of programs in Canada’s major cities provides palliative care to individuals who are precariously housed. We hope the PEACH psychiatry model can inspire the integration of psychiatric care into community-based palliative care programs for structurally vulnerable populations. Future studies should evaluate how integrated models of palliative and psychiatric care impact healthcare utilization and patient outcome measures. Qualitative studies are also needed to provide deeper insights into the experiences of both patients and providers within these integrated care frameworks. Finally, expanding education and training in this subspecialized area of psychiatry is crucial to equip professionals with the skills needed to effectively engage with and support this vulnerable population.

## Conclusion

This study describes a novel integrated psychiatry service within a community-based palliative care program for structurally vulnerable populations. The PEACH psychiatry team provides an innovative approach to care by addressing unique challenges of caring for structurally vulnerable populations.

## Supplementary Information

Supplementary Material 1.

## Data Availability

The datasets used and/or analyzed during the current study are available from the corresponding author on reasonable request.

## Abbreviations

SPMI: Severe and Persistent Mental Illness
PEACH: Palliative Education and Care for the Homeless
