# Supplementary material for: Integrating psychiatry into a community based palliative care team for structurally vulnerable populations: a descriptive retrospective cohort study of the PEACH psychiatry program model
Source: BMC Palliat Care. 2026 Jan 21;25:45. doi: 10.1186/s12904-026-01996-3 (PMC12911228; doi:10.1186/s12904-026-01996-3)
Supplement: Supplementary file 1 — Supplementary Material 1. [file 12904_2026_1996_MOESM1_ESM.docx]

Supplementary Tables

Table 4: Clients Referred to PEACH Psychiatry Team but not included in dataset

| Variable | Frequency | Percentage (n=14) | Characteristics |
| --- | --- | --- | --- |
| Referred but denied by patient | 3 | 21.4% | Declined psychiatry involvement after many discussions  Referred but declined as they had psychiatry follow up |
| Referred but not seen | 5 | 35.7% | Went to hospice/passed away prior to being seen  Not seen as being followed by another community psychiatry ACT team |
| Never formally referred but psychiatry team was consulted | 4 | 28.6% | Follow up while admitted to hospital, never officially referred  Follow up at shelter, never officially referred  Formal referral never made due to client already has a psychiatrist |
| Only seen by psychiatry | 1 | 7.1% | Client not seen by PEACH palliative care team |
| Referral Lost | 1 | 7.1% | Patient was referred and seen but the referral document could not be retrieved. |

Table 5: Additional Characterization of Substance Use and Previous Psychiatric Diagnoses of Clients Seen by PEACH Psychiatry Team

| Variable | Characteristic | Frequency |
| --- | --- | --- |
| Substance Use  (n=108) | Alcohol  Stimulants  Cigarettes/Nicotine  Polysubstance use (excluding nicotine)  Opioids  Cannabis  None  Other | 26  17  16  13  11  11  10  4 |
| Previous Psychiatric Diagnosis  (n=81) | Depression  Psychotic Disorders  Substance Use Disorders  Anxiety Disorders  None  Personality Disorders  Trauma-Related Disorders  Bipolar Disorders  Other: Attention-Deficit and Hyperactivity Disorder, Neurocognitive Disorders, Somatic Symptom and Related Disorder (FND), Hoarding Disorder (or OCD and Related Disorder) | 15  13  15  11  8  4  5  4  6 |
